# Declining in efficacy of a three-day combination regimen of mefloquine-artesunate in a multi-drug resistance area along the Thai-Myanmar border

**DOI:** 10.1186/1475-2875-9-273

**Published:** 2010-10-08

**Authors:** Kesara Na-Bangchang, Ronnatrai Ruengweerayut, Poonuch Mahamad, Kulaya Ruengweerayut, Wanna Chaijaroenkul

**Affiliations:** 1 Graduate Programme in Biomedical Sciences, Thammasat University, Thailand; 2 Mae-Sot General Hospital, Mae-Sot, Tak Province, Thailand

## Abstract

**Background:**

Declining in clinical efficacy of artesunate-mefloquine combination has been documented in areas along the eastern border (Thai-Cambodian) of Thailand. In the present study, the clinical efficacy of the three-day combination regimen of artesunate-mefloquine as first-line treatment for acute uncomplicated falciparum malaria in Thailand was monitored in an area along the western border (Thai-Myanmar) of the country.

**Methods:**

A total of 150 Burmese patients (85 males and 65 females) aged between 16 and 50 years who were attending the Mae Tao clinic, Mae-Sot, Tak Province, and presenting with symptomatic acute uncomplicated *Plasmodium falciparum *malaria were included into the study. Patients were treated initially (day 0) with 4 mg/kg body weight artesunate and 15 mg/kg body weight mefloquine. The dose regimen on day 2 was 4 mg/kg body weight artesunate and 10 mg/kg body weight mefloquine. On day 3, artesunate at the dose of 4 mg/kg body weight was given with 0.6 mg/kg body weight primaquine. Whole blood mefloquine and plasma artesunate and dihydroartemisinin (active plasma metabolite of artesunate) concentrations following treatment were determined by high performance liquid chromatography (HPLC) and liquid chromatography-mass spectrometry (LCMS), respectively.

**Results:**

Thirty-four cases had recrudescence during days 7 and 42. Five and 5 cases, respectively had reinfection with *P. falciparum *and reappearance of *Plasmodium vivax *in their peripheral blood during follow-up. The Kaplan-Meier estimate of the 42-and 28-day efficacy rates of this combination regimen were 72.58% (95% CI: 63.20-79.07%) and 83.06 (95% CI 76.14-94.40%), respectively. Parasite clearance time (PCT) and fever clearance time (FCT) were significantly prolonged in patients with treatment failure compared with those with sensitive response [median (95% CI) values for PCT 32.0 (20.0-48.0) *vs *24.0 (14.0-32.0) hr and FCT 30.0 (22.0-42.0) *vs *26.0 (18.0-36.0) hr; *p *< 0.005]. Whole blood mefloquine concentrations on days 1, 7 and 14 in patients with sensitive and recrudescence response were comparable. Although plasma concentration of dihydroartemisinin at 1 hour of treatment was significantly lower in patients with recrudescence compared with sensitive response [mean (95% CI) 456 (215-875) *vs *525 (452-599) ng/ml; *p *< 0.001], the proportion of patients with recrudescence who had relatively low (compared with the lower limit of 95% CI defined in the sensitive group) was significantly smaller than that of the sensitive group.

**Conclusions:**

Although pharmacokinetic (ethnic-related) factors including resistance of *P. falciparum *to mefloquine contribute to some treatment failure following treatment with a three-day combination regimen of artesunate-mefloquine, results suggest that artesunate resistance may be emerging at the Thai-Myanmar border.

## Background

Malaria remains a substantial public health problem in several tropical areas. The development and spreading of multidrug-resistant *Plasmodium falciparum*, particularly the previous mainstay anti-malarial drug chloroquine, sulphadoxine-pyrimethamine and mefloquine is further aggravating the situation [1]. This has complicated efforts to control the disease, and can lead to unnecessary mortality if ineffective drugs remain the standard of care after drug-resistant strains become established. To deal with the threat of resistance of *P. falciparum *to monotherapies, combinations of anti-malarials are now recommended by the World Health Organization (WHO). Artemisinin-based combination therapy (ACT) is widely promoted as a strategy to counteract the increasing resistance of *P. falciparum *to anti-malarials, as well as to prevent disease transmission and reduce the risk of drug resistance [2].

In Thailand, where the most resistant parasites are found, the National Malaria Control Programme has successfully reduced malaria morbidity and mortality in most areas of the country deploying vector control measures and a large network of malaria clinics delivering free diagnosis and treatment [3]. The number of cases has markedly decreased over the past few decades. Malaria transmission is currently limited to displaced persons and migrant workers in the forested and hilly areas of the western and eastern border provinces of Thailand along the Thai-Myanmar and Thai-Cambodian borders. Thailand was the first country to use monotherapy of mefloquine for the treatment of uncomplicated malaria, but resistance to mefloquine developed rapidly on both borders [4-6]. Mefloquine resistance led to replacement by artesunate-mefloquine in Thailand in 1995, initially as a two-day combination regimen which ensured better compliance than an extended three-day regimen of 25 mg/kg of mefloquine and 12 mg/kg of artesunate [7]. This combination regimen has led to a reduction in falciparum malaria incidence including mefloquine resistance [8]. In 2007, the malaria control programme of Thailand switched to a three-day treatment course in accordance with WHO recommendation. With the exception of the areas along the Thai-Cambodian border, this combination regimen remains effective throughout the country. Recent reports of high failure rates associated with mefloquine-artesunate combination, as well as *in vitro *drug-susceptibility data, suggest the possibility of clinical artemisinin resistance along the Thai-Cambodian border [9,10]. A considerable proportion of the malaria cases in Thailand is due to transmission occurring across both Thai-Cambodian and Thai-Myanmar borders. Substantial exchange of parasites occurs across these borders and it would, therefore, be expected that the parasite populations in both border areas are similar.

In the present study, the clinical efficacy of the three-day combination regimen of artesunate-mefloquine was investigated in a total of 150 Burmese patients who resided in an area along the Thai-Myanmar border. Resistance of *P. falciparum *strains was confirmed by the adequacy of whole blood mefloquine and plasma artesunate/dihydroartemisinin concentrations following treatment.

## Methods

### Study site and patients

The study was conducted at Mae Tao clinic for migrant workers, Tak Province during March 2008-February 2009. Malaria is a serious imported medical problem in this area with a low and stable disease transmission with two seasonal peaks and forest-related during May-August and November-January of each year. *Anopheles minimus *and *Anopheles dirus *are the principal vectors. *P. falciparum *and *Plasmodium vivax *are the two predominant species with the incidence of 1:1, *Plasmodium malariae *is occasionally found and *Plasmodium ovale *is rare. All age groups are affected and nearly all the *P. falciparum *infections are symptomatic [11].

The study was approved by the Ethics Committee of the Ministry of Public Health of Thailand. A total of 150 Burmese patients aged above 15 years who presented with typical symptoms of malaria and had a blood smear positive for *P. falciparum *were included into the study. Inclusion criteria for enrolment in the study were according to the WHO protocol for areas with low to moderate malaria transmission [12]: axillary temperature ≥37.5°C or recent history of fever; a slide-confirmed *P. falciparum *mono-infection with a parasite density 1,000-100,000 asexual parasites/*μl*. Patients with signs of severe or complicated malaria [13], febrile diseases other than malaria, presence of severe malnutrition, history of hypersensitivity reactions to any of the study drugs, and pregnant or breast-feeding women were not included. Written informed consents were obtained from all patients before study participation.

### Treatment, sample collection and follow-up

Prior to treatment (day 0), blood sample (3 ml) was collected from each patient for determination of baseline anti-malarial drug concentrations (mefloquine, artesunate and dihydroartemisinin). Patients were treated with a three-day combination regimen of artesunate and mefloquine. The initial dose of 4 mg/kg body weight artesunate (200 mg, 4 tablets of 50 mg artesunate, Atlantic Pharmaceutical Company, Thailand) and 15 mg/kg body weight mefloquine (750 mg, 3 tablets of 250 mg mefloquine; Atlantic Pharmaceutical Company, Thailand) were given on the first day (day 0). The dose regimen on day 2 was 4 mg/kg body weight artesunate (200 mg, 4 tablets of 50 mg artesunate) and 10 mg/kg body weight mefloquine (500 mg, 2 tablets of 250 mg mefloquine). On day 3, artesunate at the dose of 4 mg/kg body weight was given with 0.6 mg/kg body weight primaquine (2 tablets of 15 mg primaquine; Government Pharmaceutical Organization of Thailand). All doses were given under supervision and patients were observed for 30 minutes. If patient vomited within 30 minute, the drug dosages were repeated. When necessary, symptomatic treatment with antipyretic paracetamol, and anti-emetic dimenhydrinate was administered.

Patients were admitted to the clinic during the three-day course of treatment or until signs and synptoms of malaria disappeared. Blood samples were collected into sodium heparinized tubes at various time points after the first dose from different sub-groups of patients for determination of mefloquine (1 ml whole blood) and artesunate/dihydroartemisinin (1 ml plasma) levels. This was part of the population pharmacokinetics/pharmacodynamics study (to be described elsewhere). Plasma samples for determination of artesunate and dihydroartemisinin were separated within 30 min after collection through centrifugation at 1,200 × *g *for 15 min and stored at -180°C in a liquid nitrogen tank until analysis. Whole blood samples for mefloquine were immediately stored at -20°C until analysis.

Patients were requested to return for follow-up on days 7, 14, 21, 28 and 42 or at any time if fever or symptoms suggestive of malaria developed. At each visit, a parasite count was performed (Giemsa-stain), and a detailed questionnaire for general symptoms was recorded. Malaria blood smears were obtained on enrolment and thereafter, twice daily until two consecutive slides were confirmed to be negative, as well as at every follow-up visit. Thick films were screened for 200 oil-immersion fields before declaring a slide negative. Asexual parasites and gametocytes were separately counted against 200 WBCs; if the parasite density was too numerous to count on the thick film, the number of parasites *per *2,000 RBCs on the thin film was counted.

Patients failing to respond to the three-day regimen of artesunate-mefloquine were treated with the second-line treatment for uncomplicated falciparum malaria (quinine plus doxycycline given for 7 days). Those who developed *P. vivax *malaria in their peripheral blood during the follow-up periods were treated with 300 mg (base) of chloroquine to suppress symptoms and a full course of treatment was given at the end of the study period (chloroquine 1,500 mg given over 48 hours, followed by 15 mg (base) of primaquine daily for 14 days).

### Efficacy assessment

The clinical outcome of a 3 day course of artesunate-mefloquine was evaluated in the group of patients who completed the 42 day follow-up period. The classification of the therapeutic outcome was according to the WHO protocol [12] as 'Adequate Clinical and Parasitological Response (ACPR)', 'Early Treatment Failure (ETF)' and 'Late Treatment Failure (LTF)', which was further classified as 'Late Parasitological Failure' (LPF) or 'Late Clinical Failure (LCF)'. The primary end point was the 42-day cure rate and was defined as proportion of patients with ACPR after 42 days of follow-up. Secondary endpoint parameters included parasite clearance time (PCT), proportions of patients with clearance of parasitaemia by 24 (PCT_24hr_) and 48 (PCT_48 hr_) hours, fever clearance time (FCT), proportions of patients with clearance of fever by 24 (FCT_24hr_) and 48 (FCT_48 hr_) hours. PCT was defined as the time until the first series of negative smears occurred and FCT as the time from start of treatment until the axillary temperature decreased to below 37.5°C and remained below this temperature for the next 48 hours.

### Reinfections

In order to distinguish between reinfection and recrudescence, *P. falciparum *genotyping was performed on paired samples of *P. falciparum *DNA. Blood spots from each patient were collected on filterpaper (3 MM; Whatman, Springfield Mill, UK) and parasite variants of pre-treatment and post-treatment sample pairs were identified by using nested PCR amplification of 3 polymorphic genes for merozoite surface protein 1 (*msp1*), *msp2*, and glutamate-rich protein [14].

### Drug analysis

Concentrations of mefloquine in whole blood were measured by high performance liquid chromatography with UV-detection (HPLC-UV) according to the method of Karbwang [15], with quantification limit of 2 ng/ml. Plasma concentrations of artesunate and its active metabolite dihydroartemisinin were measured by liquid chrolatography mass-pectrometry (LCMS) according to the method of Thuy [16], with quantification limit of 2 ng/ml.

### Data analysis

Kaplan-Meier analysis was performed to calculate the proportion of parasitemic patients at each follow-up visit (days 7, 14, 21, 28, 35 and 42). Mean (95% CI) values were used to present the data which were normally-distributed continuous variables and statistical analysis for comparison was performed using analysis of variance (ANOVA), student *t*-test, or paired *t*-test, where appropriate. Median (95% CI) values were used to present the data which were not normally-distributed continuous variables and statistical analysis for comparison was performed using Kruskal Wallis, Mann-Whitney *U *test or Wilcoxon Signed Rank test, where appropriate. Difference in proportion was investigated using the Chi-squared test. Statistical significance was set at α = 0.05 for all tests.

## Results

### Clinical efficacy

**Figure 1:**
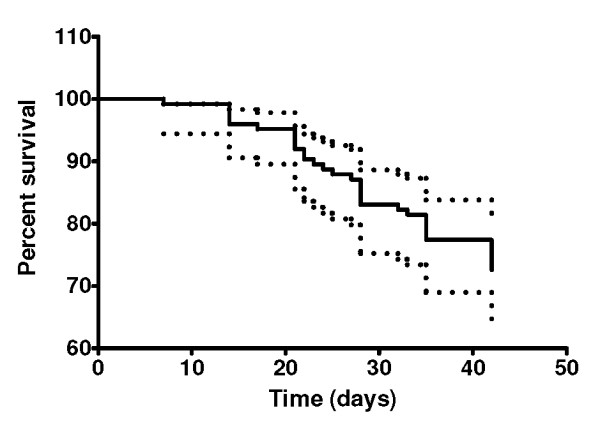
**Kaplan-Meier curve for a 42-day follow-up in 124 patients after treatment with a three-day combination regimen of artesunate-mefloquine**.

A total of 150 Burmese patients (85 males, 65 females) aged between 16 and 50 years with acute uncomplicated falciparum malaria received treatment with the first-line three-day artesunate-mefloquine combination. The treatment regimen was well tolerated; only 17 and 21 cases had nausea and headache, respectively. One hundred and thirty-two patients were evaluable for primary endpoint; 18 cases were lost to follow up during days 7 and 35. Among the 132 evaluable cases, 39 cases developed LTF, all of which were classified as LPF (recrudescence between day 7 and day 42). The median time until reemergence of parasites in the peripheral blood was 28.0 days (95% CI 7.0-42.0 days). Molecular analysis suggested that five patients had acquired a reinfection with *P. falciparum *during the 42 days follow-up (days 23, 24, 28, 35 and 42). Most treatment failures (11 cases) occurred during the fourth week of follow up. Five cases had reappearance of *P. vivax *in their peripheral blood on days 35 (2 cases) and 42 (3 cases). The probability estimate for ACPR at day 42 (42-day cure rate) and day 28 (28-day cure rate) in Kaplan-Meier analysis were 72.58% (90/124: 95% CI: 63.20-79.07%) and 83.06 (104/124: 95% CI 76.14-94.40%), respectively (Figure 1).

Patients with recrudescence (confirmed by PCR) and sensitive responses were well matched in regard to sex, age, weight, height, and parasite density on enrollment (Table 1). The PCT and FCT were significantly prolonged in patients with recrudescence compared with those with sensitive response [median (95% CI) values for PCT 32.0 (20.0-48.0) *vs *24.0 (14.0-32.0) hr; and FCT 30.0 (22.0-42.0) *vs *26.0 (18.0-36.0) hr]. In addition, the proportions of patients who had parasitaemia (PCT_24 hr_) and fever (FCT_24 hr_) cleared within 24 hours were significantly higher in the group with sensitive response (Table 1).

**Table 1:** Summary of cases included in the study and clinical response.

	Sensitive response	Recrudescence response
N	90	34

Age (yr)	25.0 (18-50)	25.0 (16.0-45.0)

Sex (n: male, female)	47, 43	15, 19

Body weight (kg)	55.0 (39.0-72.0)	52.0 (44.0-73.5)

Admission parasitemia (/ml)	5,232(1,260-84,000)	6,002(2,450-34,300)

Parasitemia on the day of recrudescence (/ml)	-	1,030(120-3,300)

Day of recrudescence (days)	-	28.0 (7.0-42.0)

PCT (hr)	24.0(14.0-32.0)	32.0(20.0-48.0)^a^

PCT_24 hr _(hr: n, %)	56, 62.2^b^	6, 17.6

PCT_48 hr _(hr: n, %)	90, 100	34, 100

FCT (hr)	26.0(18.0-36.0)	30.0(22.0-42.0)^c^

FCT_24 hr _(hr: n, %)	37, 41.1^d^	7, 20.5

FCT_48 hr _(hr: n, %)	90, 100	34, 100

Data are presented as median (95% CI) or proportion (n, %)

^a^= Statistically significantly longer than patients with sensitive response (Mann Whitney U-test, p < 0.005)

^b^= Statistically significantly higher than patients with recrudescence response (Chi-square test, p < 0.01)

^c^= Statistically significantly longer than patients with sensitive response (Mann Whitney U-test, p < 0.005)

^d^= Statistically significantly higher than patients with recrudescence response (Chi-square test, p < 0.01)

### Drug concentrations

**Figure 2:**
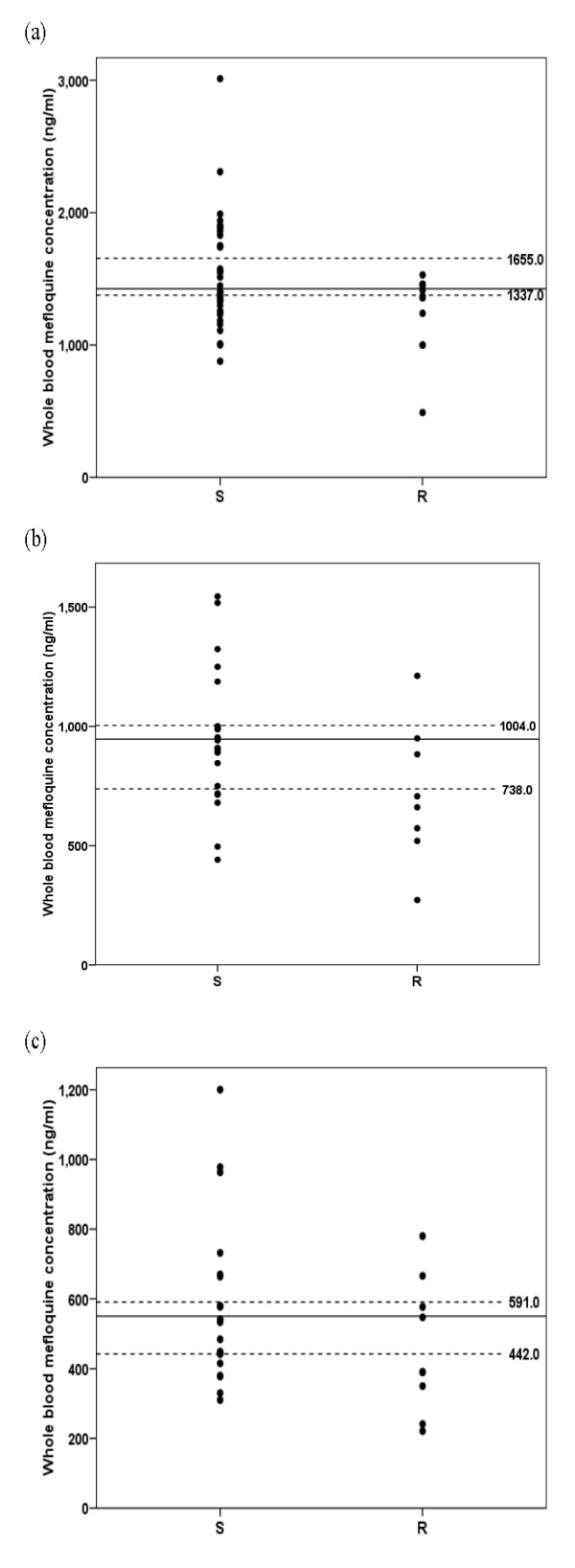
**Whole blood mefloquine concentrations on day 1 (a), day 7 (b) and day 14 (c) after treatment in patients with sensitive (S) and recrudescence (R) response**. Dot lines represent the lower and upper level of 95% CI defined in the sensitive group.

**Figure 3:**
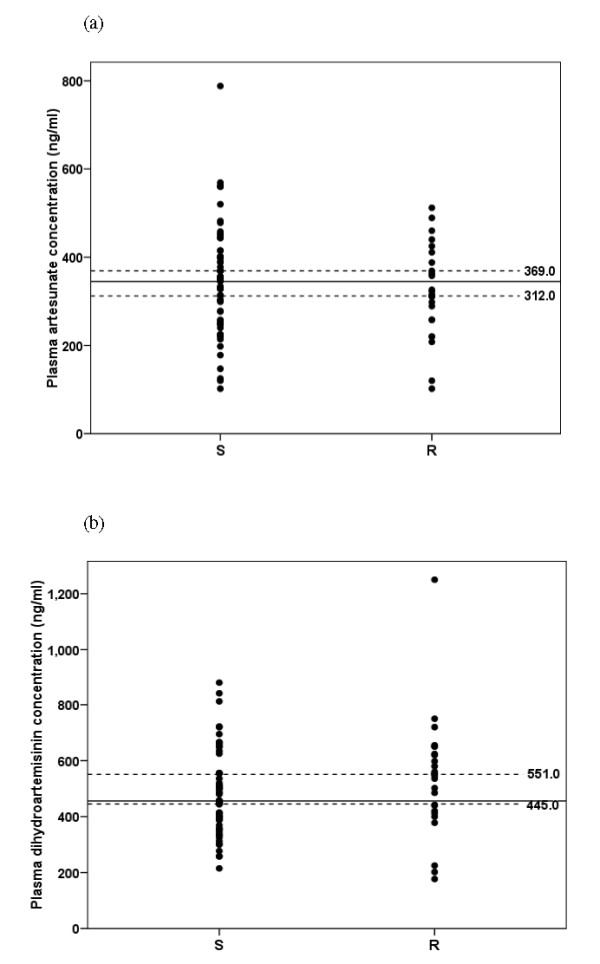
**Plasma artesunate (a) and dihydroartemisinin (b) concentrations at 1 hour of the first dose in day 1 in patients with sensitive (S) and recrudescence (R) response**. Dot lines represent the lower and upper level of 95% CI defined in the sensitive group.

Whole blood mefloquine concentrations on days 0 (pre-treatment), 1, 7 and 14 of treatment are summarized in Table 2 and Figure 2 and plasma artesunate and dihydroartemisinin at 0 (pre-treatment) 1 and 6 hour after the first dose are summarized in Table 3 and Figure 3. Baseline mefloquine concentrations were detected in 15 (11.36%) cases (11 and 4 cases in the group with sensitive and recrudescence response, respectively) with median (95% CI) of 0 (0-220) ng/ml. No measurable baseline artesunate or dihydroartemisinin was found. Whole blood mefloquine concentrations tended to be lower on days 1, 7, 14 and 21 of treatment in patients with recrudescence response (Table 2). However, due to large inter-individual variability, statistical significance was not reached. Patients who had reappearance of parasitaemia on day 7 (1 case) or day 42 (5 cases) had similar mefloquine concentrations on days 1, 7 and 14. Nevertheless, the proportion of patients in the recrudescence group who had mefloquine concentrations on days 1, 7 and 14 lower than the lower limit of 95% CI for median concentrations defined in the sensitive group (1,377, 738 and 442 ng/ml for days 1, 7 and 14, respectively) was significantly larger than the sensitive group [5 (41.7%) *vs *9 (25%), 3 (33.3%) *vs *2 (9%) and 5 (50%) *vs *5 (23.8%) cases for concentrations on day 1, 7 and 14, respectively] (Figure 2). Plasma aretsunate concentrations at 1 and 6 hours after the first dose were comparable between the group with recrudescence and sensitive response. On the other hand, dihydroartemisinin concentrations at 1 hour of treatment was significantly lower in patients with recrudescence response [mean (95% CI) 456 (215-875) *vs *525 (452-599) ng/ml]. For both artesunate and dihydroartemisinin, the proportions of patients in the recrudescence group who had the concentrations at 1 hour after the first dose lower than the lower limit of 95% CI for median concentrations defined in the sensitive group (312 and 445 ng/ml for artesunate and dihydroartemisinin, respectively) was significantly smaller than the sensitive group [8 (26.6%) *vs *26 (40%) and 10 (33.3%) *vs *30 (46.1%) for artesunate and dihydroartemisinin, respectively] (Figure 3). Among the 34 cases with recrudescence, 12 had adequate blood/plasma concentrations of both mefloquine and dihydroartemisinin. Inadequacy of mefloquine and dihydroartemisinin concentrations was observed in 13 and 10 cases with recrudescence, respectively. Inadequate concentrations of both drugs was found in one case.

**Table 2:** Whole blood concentrations of mefloquine on Day-0 (pre-treatment), days 7, 14, 21, 28, 35 and 42 of treatment in patients with sensitive response and recrudescence.

Day of treatment	Sensitive response		Recrudescence response	
	
	N	Mean (95%CI)* or Median (95% CI)**	N	Mean (95%CI)* or Median (95% CI)**
Day 0**	90	0 (0-1,714)	36	0 (0-92)

Day 1*	36	1,411 (1,216-1,605)	12	1,345 (1,067-1,624)

Day 7*	22	1,055 (806-1,304)	9	754 (541-968)

Day 14*	21	659 (426-893)	10	421 (181-660)

Day 21*	15	416 (258-575)	11	359 (230-488)

Day 28*	13	233 (158-307)	8	288 (146-329)

Day 35*	12	145 (96-195)	6	179 (41-317)

Day 42**	19	19 (5-163)	9	13 (0-49)

**Table 3:** Plasma concentrations of artesunate and dihydroartemisinin on day-0 (pre-treatment) and at 1 and 6 hours after treatment in patients with sensitive response and recrudescence.

Drug/Day of treatment	Sensitive response		Recrudescence response	
	
	N	Mean (95%CI)* or Median (95% CI)**	N	Mean (95%CI)* or Median (95% CI)**
*Artesunate:*				

Day 0**	90	0	35	0

Hour 1*	65	344 (312-375)	30	323 (282-364)

Hour 6**	64	1 (0-1,253)	30	0 (0-12)

*Dihydroartemisinin:*				

Day 0**	90	0 (0-10)	35	0

Hour 1*	65	525 (452-599)	30	456 (215-875)^a^

Hour 6**	64	98 (13-220)	30	100 (0-220)

^a ^= Statistically significantly lower than patients with sensitive response (student t-test; p < 0.001)

## Discussion

An artesunate-mefloquine combination (with mefloquine doses varying from 750 to 1,250 mg depending on endemic areas) given in three days has become the first-line treatment for falciparum malaria in Thailand since 2005 with satisfactory clinical efficacy and tolerability. In the present study however, it is noted for an unexpectedly high failure rate for this three-day combination for treatment of uncomplicated falciparum malaria in the area along the Thai-Myanmar border. The cure rates at 28 and 42 days, calculated by Kaplan-Meier survival analysis and PCR correction for re-infection, were 83.06 and 72.58%, respectively. It is noted that reappearance of parasitaemia occurred as early as 7 days till 42 days after the first dose. In addition, there was a small but significant delay of parasite clearance in the group with recrudescence response [median (range) PCT 32.0 (20.0-48.0) hours] compared to the sensitive group [24.0 (14.0-32.0) hours]. Only 6 (17.6%) and 7 (20.5%) patients with recrudescence response respectively, had parasitaemia (PCT_24 hr_) and fever (FCT_24 hr_) cleared within 24 hours. The efficacy of this combination was markedly dropped when compared with our recent investigation during 2008-2009 with the same regimen in the same area, where a 42-day cure rate of 99.2% was obtained [17]. In other studies conducted in the same area reported during 1997-2003, a 28 or 42 day cure rates [18-20] for artesunate-mefloquine combination were also approaching 100%. In the areas of Thai-Cambodian border (Ranong, Chantaburi Provinces) with highly mefloquine resistance except Trad Province (78.6%), cure rates were greater than 90% [9]. The high failure rate observed in the present study was not due to compliance factor since all patients were admitted in the clinic during the three-day course of treatment. Mefloquine baseline level ranging from 9-300 ng/ml detected in about 11.3% would imply previous treatment with mefloquine for the previous/current malaria episode. In our previous study, compliance with this three-day combination regimen conducted in malaria clinics under the Malaria Control Program of Thailand was also high (96.3%) and baseline mefloquine levels were observed in 24% [17].

'Genuine resistance' of treatment failure cases was confirmed by monitoring for the adequacy of whole blood mefloquine and plasma artesunate/dihydroartemisinin concentrations. Mefloquine concentrations on days 1, 7 and 14 of treatment in patients with recrudescence response tended to be lower than those with sensitive response and with larger proportion of cases with the concentrations (on days 1, 7 and 14) lower than the lower limit of 95% CI defined in the sensitive group. Blood concentrations of mefloquine on day 1 of treatment has been reported to be an important determinant of successful treatment [21]. On the other hand, although dihydroartemisinin concentrations were significantly lower in patients with recrudescence, the smaller proportion of patients in the recrudescence group had the concentrations at 1 hour of treatment of both artesunate and dihydroartemisinin lower than the threshold levels compared with the sensitive group (8 *vs *26% and 10 *vs *30%, respectively). Dihydroartemisinin is the active plasma metabolite of artesunate, which has a longer half-life (1-2 hours) with potency 2-4 times greater than the parent drug [22]. This suggests that despite the relatively low dihydroartemisinin concentration at 1 hour (< 445 ng/ml) in about 33% (10 cases) of patients with recrudescence response, they were in most cases adequately kill *P. falciparum *isolates in this area. There has been no defined threshold levels of artesunate/dihydroartemisinin for treatment of falciparum malaria but it is noted however that artesunate concentration (at 1 hour) of as low as 120 ng/ml was adequate in two patients with sensitive response, while dihydroartemisinin concentration (at 1 hour) of as high as 1,250 ng/ml was inadequate in one case with recrudescence. The later had also confirmed adequate mefloquine concentrations on days 1, 7 and 14. While a total of 12 patients had confirmed adequacy of whole blood/plasma concentrations of both mefloquine and dihydroartemisinin, in adequacy of the concentrations of mefloquine alone, dihydroartemisinin alone and both contributed to 13, 10 and one cases with treatment failure, respectively. None of the treatment failure vomited the doses of mefloquine or artesunate. It was reported that patients from Tak Province experiences a lower frequency of nausea and vomiting than other groups [9].

Results of clinical efficacy together with drug concentration profiles may suggest the possibility of contribution of pharmacokinetic factors as well as mefloquine and artesunate/dihydroartemisinin resistance to the observed high failure rate of the current three-day combination regimen. With the absence of *in vitro *sensitivity data of individual parasite isolate, it is difficult to clearly define whether reduced clinical efficacy of the combination is due to which of each partner drug. It is noted however that mefloquine concentration profiles in this patient population (Burmese) appear lower (by about 30%) than our previous reports in Thai patients following the same dose of mefloquine [17,23]. The relatively low mefloquine concentrations in the recrudescence cases may be due to variability in pharmacokinetics and the levels were no longer adequate once the level of mefloquine resistance is aggravating. Furthermore, it also appears possible that artesunate resistance is beginning to emerge in this area on a background of pre-existing mefloquine resistance. Solely mefloquine resistance should not significantly affect the parasite clearance time following treatment with artesunate-mefloquine combination. However, with a good malaria control in this area, the contribution of low immunity to poor clinical response cannot be excluded. Mefloquine was used as mono-therapy for treatment of acute uncomplicated falciparum in this area long before the introduction of the combination regimen and thus mefloquine resistance in this area had already reached a level too extreme to protect the development and spread of artesunate resistance. The high proportion of patients with baseline mefloquine concentrations points to the high selective drug pressure in this area. The dramatic drop in clinical efficacy of the three-day artesunate-mefloquine combination after 13 years continuous deployment in this area is alarming and is of concern as that has occurred in the Thai-Cambodian border and containment measures are urgently needed. In the Thai-Cambodian border, resistance was characterized by a markedly prolonged time to parasite clearance, without corresponding reductions on conventional *in vitro *susceptibility testing, pharmacokinetic or other host factors [24]. Analysis of *in vitro *susceptibility of the parasite isolates (baseline and the day of recrudescence) together with candidate molecular markers of resistance (amplification and SNPs of *pfmdr1 *and *pfatp6*) are under way, to clearly define, with support of drug concentration data, if the reduced artesunate-mefloquine efficacy observed is really due to a true decline in artesunate sensitivity or a further progression of mefloquine resistance or both [25,26]. Of particular concern is the increased transmissibility of these tolerant parasites. Close monitoring of the *in vitro *susceptibility of *P. falciparum *to mefloquine and artesunate/dihydroartemisinin in this area, and in particular vigilance to detect early emergence of higher levels of artemisinin resistance is required

## Conclusions

The present study was the first observation of a marked decline in clinical efficacy of a three-day artesunate-mefloquine regimen in the area along the Thai-Myanmar border. Although pharmacokinetic (ethnic-related) factors including resistance of the parasite to mefloquine mainly contribute to some treatment failure, results suggest that artesunate resistance may be emerging at the Thai-Myanmar border.

## Competing interests

The authors declare that they have no competing interests.

## Authors' contributions

All authors read and approved the final manuscript.
